# Microneedle-Assisted Delivery of Curcumin: Evaluating the Effects of Needle Length and Formulation

**DOI:** 10.3390/mi16020155

**Published:** 2025-01-29

**Authors:** Em-on Chaiprateep, Soma Sengupta, Cornelia M. Keck

**Affiliations:** 1 Department of Pharmaceutics and Biopharmaceutics, Philipps-Universität Marburg, Robert-Koch-Str. 4, 35037 Marburg, Germany; emon_c@rmutt.ac.th (E.-o.C.); sengupts@pharmazie.uni-marburg.de (S.S.); 2 Faculty of Integrative Medicine, Rajamangala University of Technology Thanyaburi (RMUTT), Thanyaburi 12130, Thailand

**Keywords:** microneedles, dermaroller, nanocrystals, bulk suspension, derma pens, dermal penetration, curcumin, particle size

## Abstract

Dermal drug delivery presents a significant challenge for poorly soluble active compounds like curcumin, which often struggle to penetrate the skin barrier effectively. In this study, the dermal penetration efficacy of curcumin nanocrystals and bulk suspensions when applied to skin using microneedles of varying lengths—0.25 mm, 0.5 mm, and 1.0 mm—was investigated in an ex vivo porcine ear model. The findings revealed that all formulations, in conjunction with microneedle application, facilitated transepidermal penetration; however, the combination of microneedles and curcumin nanocrystals demonstrated the highest efficacy. Notably, the 1.0 mm microneedle length provided optimal penetration, significantly enhancing curcumin delivery compared with bulk suspensions alone. Additionally, even the use of 0.25 mm microneedles resulted in a high level of efficiency, indicating that shorter microneedles can still effectively facilitate drug delivery. Overall, this study underscores the potential of microneedle technology in improving the transepidermal absorption of poorly soluble actives like curcumin, suggesting that the integration of nanocrystals with microneedles could enhance the therapeutic effects of topical curcumin applications.

## 1. Introduction

Microneedles are minimally invasive devices that have gained significant attention in the field of transdermal drug delivery. These small, needle-like structures, typically ranging from 25 to 1000 μm in length, are designed to penetrate the outer layer of the skin (stratum corneum) without reaching the nerve endings, thereby minimizing pain and discomfort [1].

The mechanism of action for microneedles involves creating micro-scale punctures in the skin, which enhances its permeability and allows for the efficient delivery of macromolecules such as peptides, proteins, and vaccines that are generally unable to penetrate the skin through traditional methods [2]. Microneedles can be fabricated from various materials, including polymers, metals, and ceramics, and are designed for a range of applications, from vaccination to chronic disease management [3].

Microneedles can be classified into several categories based on their design and application. Solid microneedles are primarily used for drug delivery, creating microchannels in the skin without containing drugs themselves. Dissolvable microneedles, on the other hand, are composed of a drug–polymer matrix that dissolves upon insertion into the skin, releasing the drug directly into the tissue. Coated microneedles feature solid needles coated with a drug formulation, which is delivered as the needle is inserted and withdrawn. Hydrogel microneedles swell upon contact with the skin, enhancing drug release and absorption [4].

The advantages of microneedle technology are notable. The small size of these devices minimizes pain, making them more acceptable to patients compared with hypodermic needles [5,6,7]. Additionally, microneedles can be self-administered, which enhances adherence to treatment regimens, particularly for vaccines and chronic disease therapies [8,9]. Furthermore, the ability to design microneedles for controlled release allows for sustained drug delivery, improving therapeutic outcomes [10,11,12,13].

Despite the promising applications of microneedles, several challenges remain. Concerns about the stability of drug formulations, manufacturing scalability of microneedle arrays, and regulatory hurdles need to be addressed [14,15,16]. Future research is focused on overcoming these challenges and aims at further improving the use and application of this technique.

Recent advancements have also used and integrated nanoparticles with microneedles to further enhance drug delivery. Nanoparticles can be loaded with therapeutic agents and then incorporated into microneedle systems, providing a platform for controlled and sustained release [17]. This combination can improve the bioavailability of poorly soluble drugs and facilitate the delivery of a wider range of therapeutic agents, including nucleic acids and anticancer drugs [18,19,20].

Most nanocarriers are used to encapsulate the drug. Hence, the drug is loaded in a vehicle, which means the drug loading capacity is below 100%, and transdermal delivery of these formulations would mean that not only the drug but also the material of the carrier/vehicle is transported into the skin. If the material of the carrier/vehicle is not skin-friendly, this might cause undesired side effects. Therefore, to circumvent this, a nanocarrier that is composed of 100% active ingredients would be optimal.

Nanocrystals are crystalline particles with dimensions typically in the range of 1–1000 nanometers. Their small size increases the surface area-to-volume ratio, which can enhance the solubility and bioavailability of poorly soluble drugs. When incorporated into transdermal formulations, nanocrystals can facilitate better penetration through the skin barrier. They can be used in various forms, such as suspensions or incorporated into gels and creams.

Studies have demonstrated that nanocrystals can significantly improve the skin permeation of drugs. For instance, it was reported that the incorporation of carvedilol nanocrystals into a gel formulation increased drug permeation through human skin by 2.5-fold compared with conventional formulations [21]. This enhanced delivery is attributed to the reduced particle size, which allows for better solubility of the active material, improved penetration, and improved interaction of the particles with the skin.

Consequently, combining nanocrystals and microneedles can lead to synergistic effects, enhancing drug delivery even further. For instance, microneedles can be coated with drug-loaded nanocrystals, allowing for immediate release upon insertion into the skin. This combination not only improves the solubility and bioavailability of the drug but also ensures that the drug reaches deeper dermal layers, where it can exert its therapeutic effects. Recent research has shown that this combination can significantly enhance therapeutic outcomes [22,23,24,25,26,27,28,29]. However, a detailed study investigating the effect of microneedle device type, needle length, and particle size on the dermal penetration efficacy of active ingredients is not yet available. Since these details are important for the thoughtful selection of a microneedle device, this study was performed to provide this information.

## 2. Materials and Methods

### 2.1. Materials

**Figure 1:**
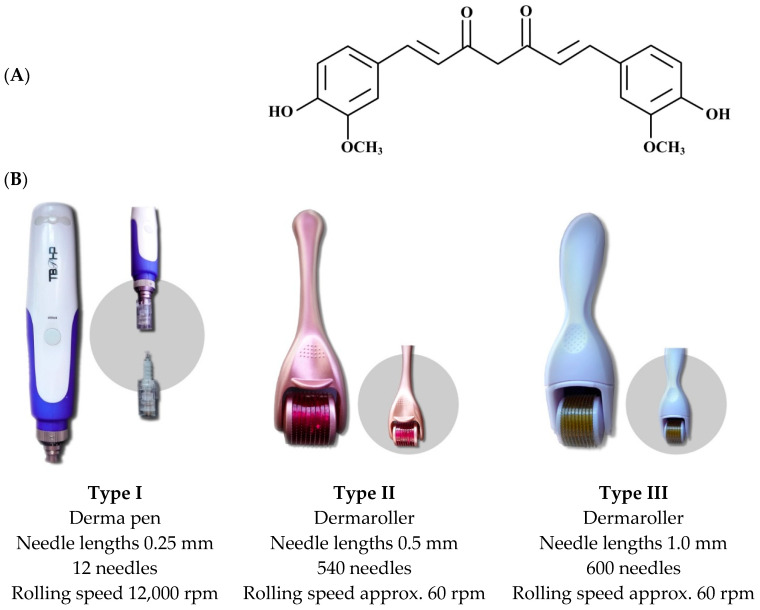
Chemical structure of curcumin (**A**) and overview of microneedle devices used in the study (**B**).

Curcumin (Figure 1A) is a poorly water-soluble compound and was used as a model drug in this study. The raw material of curcumin bulk powder was purchased from Receptura international compounding pharmacy (Frankfurt am Main, Germany). Vitamin E TPGS (D-α-tocopheryl polyethyleneglycol 1000 succinate) (GPF Company-Parmentier GmbH, Frankfurt am Main, Germany) was used as a stabilizer. The Flex 2 PURELAB^®^ (ELGA LabWater, High Wycombe, UK) was used to produce purified water freshly in-house.

### 2.2. Methods

#### 2.2.1. Production of Curcumin Bulk and Nanocrystals Suspensions

The bulk suspensions were prepared using 5% curcumin raw powder and 1% TPGS (all % *w*/*w*). The nanosuspensions were formulated by using small-scale bead milling after a previously established protocol [30,31]. For this, 10 mL of the bulk suspension was placed in a 25 mL Erlenmeyer flask to which yttrium/zirconium oxide stabilized ceramic beads (SiLibeads^®^ 9911-Ph, 1.0–1.2 mm, Sigmund Lindner, Warmensteinach, Germany) and a magnetic stirring bar with a triangular shape (Asteroid^®^ 25, 14 × 25 mm, Ø 16 mm, 2 mag AG, Hamburg, Germany) were added. The ratio of beads to curcumin solution was 40:60 (*v*/*v*). The flask was placed in an ice bath on top of a magnetic stirrer and stirred at 1500 rpm for 14 h, with a temperature not exceeding 20 °C [32]. Curcumin bulk and nanocrystal suspensions were diluted to obtain final formulations that contained 0.625% *w*/*w* curcumin.

#### 2.2.2. Physicochemical Characterization of Curcumin Bulk and Nanocrystal Suspensions

The curcumin bulk suspensions were characterized using laser diffractometry (LD) and light microscopy (LM). The curcumin nanosuspensions were characterized using LD, LM, photon correlation spectroscopy (PCS), and laser Doppler anemometry (LDA). For bulk suspension analysis, PCS and LDA were not possible due to the limitations of measuring size ranges from 1 nm to 10 μm [33].

PCS, also known as dynamic light scattering (DLS), was used to analyze the particle size (z-average) and width of the size distribution (polydispersity index, PdI) using a Zetasizer Nano-Series (Malvern Panalytical GmbH, Kassel, Germany). Samples were diluted in water, and the mean values from 10 measurements were calculated.

The LD measurements were performed for the detection of possible larger particles that, for example, could have remained after the nanomilling process. For the measurements, a Mastersizer 3000 (Malvern Panalytical GmbH, Germany) was used. Samples were analyzed using Mie theory with a refractive index (RI) of 1.87 and an imaginary refractive index (IRI) of 0.01 (red light) and 0.1 (blue light) for curcumin. The results were represented as volume-weighted diameters: d(v) 10, 50, 90, 95, and 99%.

LM was conducted using an Olympus BX53M (BX3M series, Olympus Cooperation Industrial Microscope, Tokyo, Japan).

LDA measurements were performed to assess the zeta potential (ZP) of the curcumin suspensions using a Zetasizer Nano ZS (Malvern Panalytical GmbH, Kassel, Germany). Measurements were conducted in the original dispersion medium and in water adjusted to a conductivity of 50 μS/cm with NaCl solution. LDA measurements assessed the electrophoretic mobility of the particles, which was converted into the ZP using the Helmholtz-Smoluchowski equation.

#### 2.2.3. Dermal Penetration Efficacy

The dermal penetration efficacy of curcumin from the different formulations and the influence of the different types of microneedle devices were assessed by using an ex vivo porcine ear model with subsequent digital image analysis after previously established protocols [34,35,36]. Fresh porcine ears were obtained from the local slaughterhouse and immediately used for the study within 3 h after slaughter. Porcine ears were cleaned with lukewarm water and gently dabbed dry using a soft tissue. The dorsal side of the porcine ears (outer side of the ear) was used for the study, and areas (2 × 2 cm) without visible lesions and scratches were marked. Within these areas, hairs were carefully trimmed to a length of about 2–3 mm with scissors.

Three different types of microneedles were used in this study (Figure 1B). The selection of the devices was based on the knowledge that the use of dermarollers with a length of up to 0.5 mm is considered to be painless, whereas longer needle lengths are considered to create some pain but higher [37]. Moreover, it can be considered that the use of an automatic pen might be more convenient than a manual dermaroller. The aim of this study was to investigate which type of device would lead to the best dermal penetration and is most convenient to use at the same time. Type I was an automated derma pen with 12 fine needles that punctured the skin with a vibrating stamp. The vibration speed of the derma pen was set to 1200 cycles/min (blue light), and the needle length was adjusted to 0.25 mm. Type II and III were manual dermarollers with different needle lengths and numbers of needles (Type II: 0.5 mm with 540 fine needles; Type III: 1.0 mm with 600 fine needles).

Prior to the use of the microneedling devices, 10 µL of the curcumin bulk and nanocrystal suspensions (0.625% *w*/*w*) was applied to the marked porcine skin areas. The different microneedling devices were rolled or stamped in four directions (vertical, horizontal, oblique) for 30 s by the same person at a constant applied pressure. The rolling speed of the manual devices was kept constant at about 60 rpm (approximately 1 round per second). As controls, untreated skin and skin treated with the curcumin formulations without the use of the microneedle devices were used.

The treated pig ears were incubated for 4 h at 32 °C. After this, the ears were carefully washed for 30 s in each area. Porcine ears were then carefully dabbed dry with a soft face paper towel. Each test area was then punched (15 mm drive punch), and the punch biopsies obtained were embedded in Tissue-Tek^®^ (O.C.T.^®^, Alphen aan den Rijn, The Netherlands), frozen at −40 °C, and then kept at −18 °C until further use. All experiments were performed in triplicate and on independent ears, i.e., ears from different donors.

Subsequently, the frozen punch biopsies were cut into 20 μm thick vertical skin cross-sections using a cryomicrotome (2700-Frigocut, Reichert-Jung, Wetzlar, Germany). The skin cross-sections obtained were carefully placed on objective slides and then analyzed by epifluorescence microscopy (Olympus CKX53 with an Olympus DP22 color camera, Olympus Deutschland GmbH, Hamburg, Germany). The selected fluorescence filter for the analysis was the DAPI HC filter block system (excitation filter: 460–500 nm, dichroic mirror: 500 nm, emission filter: from 500 nm (LP)). The fluorescence intensity, exposure time, and magnification were set and kept constant at 50%, 50 ms, and 200-fold magnification, respectively.

In the last step, the microscopic images obtained were analyzed using ImageJ Software version 1.53 to determine the amount of penetrated curcumin and the depth of its penetration into the skin [35,38]. The amount of penetrated curcumin was assessed by subjecting the images to an automated RGB threshold macro (Supplementary Material Table S1). The RGB macro splits the image into two parts and subtracts the autofluorescence of the skin. Therefore, the remaining number of pixels within the picture after the RGB-threshold macro (ART) can be measured as the mean grey value per pixel (MGV/px) and represents the amount of penetrated active compound into the skin [36]. From the ART picture, the mean penetration depth (MPD) was determined by measuring the distance between the upper stratum corneum and the most distant pixel in each picture. For this, the manual scale function of the software was used, and the scale was set to 2.84 pixels/µm. Similar to the MPD, the stratum corneum thickness (SCT) was analyzed. The SCT is a measure of skin hydration and was determined from the original images. For each tested skin area, at least 50 images were obtained for each ear. This means that from each tested formulation, at least 150 images were obtained and used for analysis.

#### 2.2.4. Statistical Analysis

Descriptive statistics and the comparison of the mean values were performed with JASP software (version 0.14.0). For the comparison of the mean values, normal distribution and variance homogeneity were tested with the Shapiro–Wilk and Levene’s test, respectively. One-way ANOVA was performed for normally distributed data sets, and Kruskal–Wallis tests were conducted for the non-parametric data. Games–Howell or Dunn’s post hoc tests were performed to compare the mean values between each other. Significant differences were defined as *p* < 0.05.

## 3. Results and Discussion

### 3.1. Production and Characterization of Curcumin Bulk and Nanocrystals

**Figure 2:**
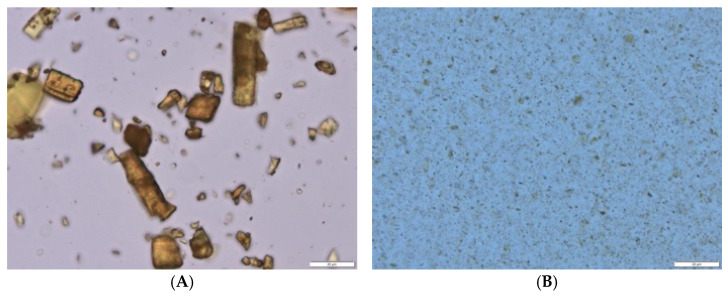
Microscopic images of curcumin suspensions: (**A**) bulk suspension and (**B**) nanocrystals (magnification 1000×, scale bar represents 20 μm).

A larger-sized curcumin bulk suspension and a small-sized curcumin nanosuspension were successfully produced in the first part of the study (Figure 2, Table 1). The curcumin bulk suspension contained particles with a mean size of about 19 μm. After bead milling, the particles had a size of about 256 nm and a PDI of 0.31 (Table 1). A PDI above 0.3 indicates a rather broad size distribution, and LD and LM analysis confirmed that the nanosuspension contained not only small-sized nanoparticles but also some agglomerates and larger-sized particles of up to 3.8 μm (Table 1 and Figure 2).

The ZP of the curcumin suspensions was about −11 mV in water and approximately −10 mV in the original dispersion medium. TPGS is a non-ionic surfactant and, therefore, creates steric stabilization for the particles. Optimal steric stabilization is considered to be achieved if the particles possess a neutral charge, i.e., a ZP of around 0 mV. The ZP of the suspension was slightly higher, indicating that the steric stabilization achieved by TPGS was not optimal for the stabilization of the particles. The ZP measurements, therefore, explain the observed slight tendency of agglomeration for some of the particles. However, the small changes between the ZP measured in water and the OM (1% TPGS solution) indicate that TPGS is tightly bound to the surface of the particles and cannot be easily washed off after the suspension is diluted in water. This means that the steric stabilization provided by the TPGS is permanent and robust and that the observed agglomeration of the nanoparticles is rather weak [39]. Hence, the particles can be easily disaggregated by shaking, which will then result in small-sized nanoparticles. This was confirmed by the LD measurements taken while the samples were stirred, which showed that 90% of the volume of the particles possesses a size of <620 nm (Table 1).

The pores in the skin that are created by microneedle devices are considered to be in the range of about 10 μm [40]. Therefore, the particles of the bulk suspensions were considered not to penetrate the holes created by the microneedles, whereas the nanocrystals, which are well below the 10 μm range, were considered capable of penetrating the pores created by the microneedle devices. The next step investigated this hypothesis and the effect of the different microneedle devices on the dermal penetration efficacy of curcumin.

### 3.2. Influence of Particle Size on Dermal Penetration Efficacy of Curcumin

**Figure 3:**
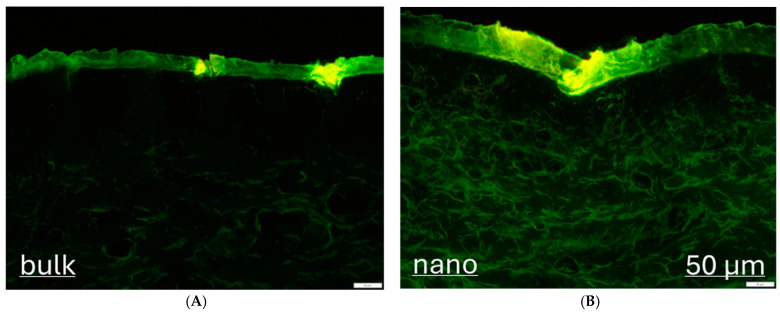
Microscopic images of vertical skin cuts treated with curcumin suspensions: (**A**) bulk suspension and (**B**) nanocrystals (magnification 200×, scale bar represents 50 μm).

**Figure 4:**
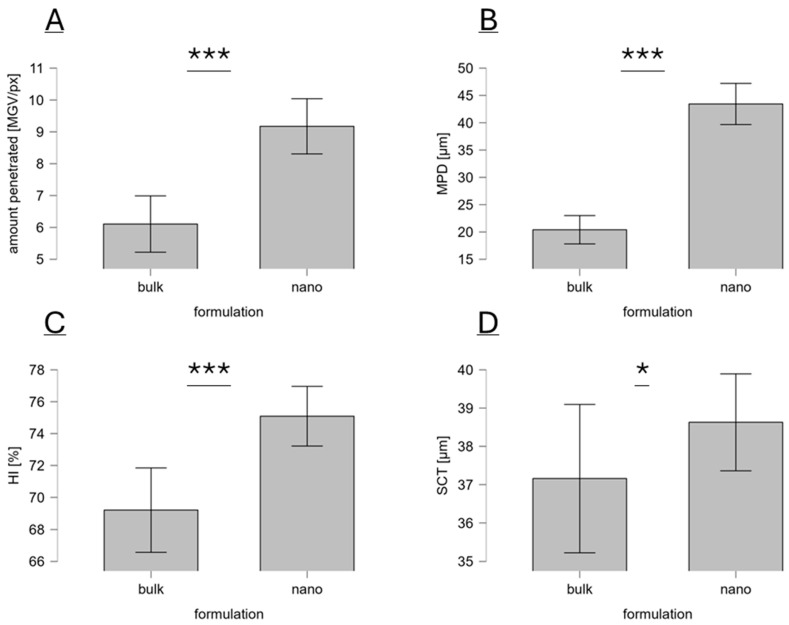
Dermal penetration efficacy of curcumin treated with curcumin bulk and nanosuspensions: (**A**) amount of penetrated curcumin, (**B**) mean penetration depth, (**C**) homogeneity index (HI), and (**D**) stratum corneum thickness. * *p* < 0.05, *** *p* < 0.001.

Treatment of the skin with the curcumin bulk and nanosuspension without subsequent microneedle treatment showed different penetration profiles for curcumin from the differently sized formulations. When compared with the bulk material, the amount of penetrated curcumin was about 1.5-fold higher, and the penetration depth was about 2-fold higher (Figure 3A,B and Figure 4A). This result is in line with previous studies and the theory of nanocrystals, which are exploited to increase the solubility and bioavailability of poorly soluble active compounds. The homogeneity index was increased by about 10%, indicating that the nanocrystals create a more homogeneous dermal penetration for curcumin than the bulk material (Figure 4C). This effect was also expected because the small-sized curcumin crystals create a more homogeneous distribution of curcumin in the formulation. After dermal application, the curcumin can, therefore, penetrate more homogeneously from this formulation than from the larger-sized formulation, where the curcumin particles are much bigger and less homogeneously distributed within the formulation (cf. Figure 2).

The SCT of the curcumin-treated skin was about 40 μm and was slightly thicker when the skin was treated with the nanosuspension (Figure 4D). The stratum corneum thickness is a measure of the skin hydration. Hence, the nanocrystal formulation caused a slightly higher skin hydration than the bulk suspension. This effect can be explained by the hygroscopic properties of curcumin. Penetrated curcumin moisturizes the skin. Therefore, the higher amount of penetrated curcumin from the nanosuspensions also caused slightly higher skin hydration (approx. +15%). The skin hydrating effect of curcumin was already seen in a previous study [41]. Therefore, the results obtained in this study confirm the skin moisturizing effect of curcumin.

**Figure 5:**
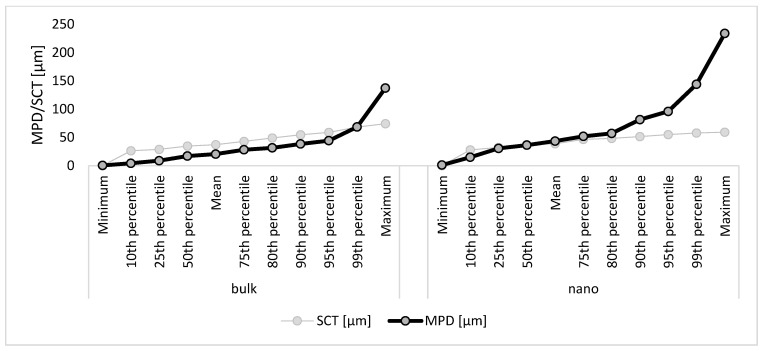
Comparison of SCT and MPD for skin treated with curcumin bulk material (**left**) and curcumin nanosuspensions (**right**), allowing for an evaluation of the percentage of how much curcumin penetrated through the stratum corneum from the different formulations.

The MPD of curcumin from the bulk material was about 20 μm and was about 43 μm from the nanocrystals (Figure 4B). When compared with the SCT, this means that the bulk suspension delivered the curcumin into the stratum corneum but not deeper (MPD < SCT). The nanocrystals were able to deliver the curcumin through the stratum corneum (MPD > SCT). More detailed information on how much curcumin was delivered through the stratum corneum is possible by comparing the percentiles of SCT and MPD (Figure 5). The results show that less than 1% of the skin biopsy images showed curcumin that penetrated through the stratum corneum when the skin was treated with the curcumin bulk suspension, but that curcumin penetrated through the stratum corneum in more than 50% of the cases when the skin was treated with the curcumin nanosuspension.

### 3.3. Influence of Type of Microneedle Device on Dermal Penetration Efficacy of Curcumin

**Figure 6:**
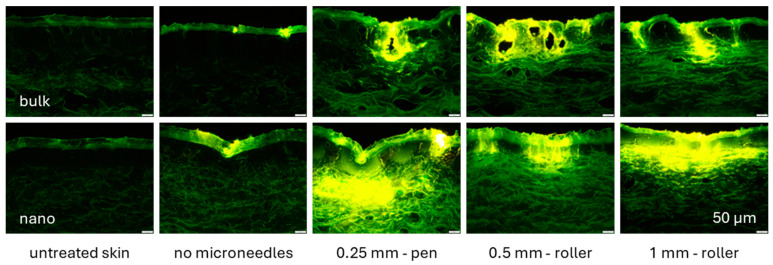
Microscopic images of skin biopsies showing the influence of the type of microneedle device on dermal penetration efficacy of curcumin from bulk suspensions (**upper**) and nanosuspensions (**lower**).

**Figure 7:**
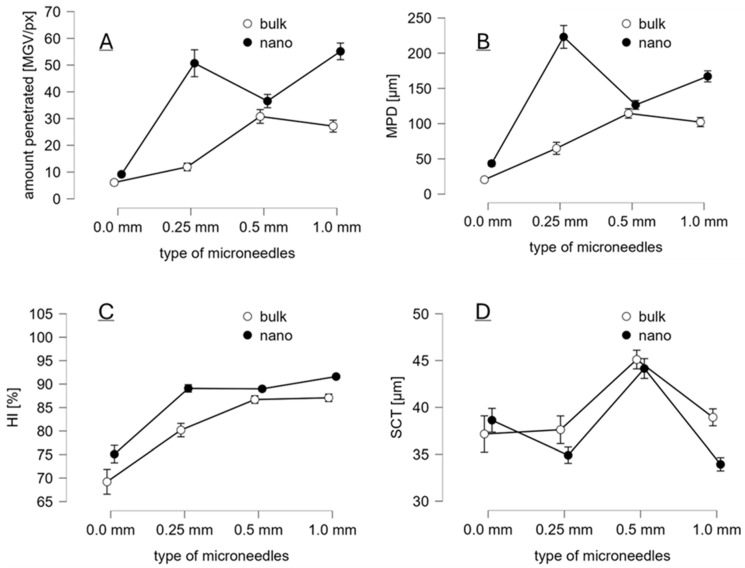
Dermal penetration efficacy of curcumin treated with curcumin bulk and nanosuspensions and with different types of microneedle devices, where (**A**) amount of penetrated curcumin, (**B**) mean penetration depth, (**C**) homogeneity index (HI), and (**D**) stratum corneum thickness (SCT). A 0.0 mm type of microneedle refers to skin that was treated with the curcumin formulations but was left without microneedle treatment.

Treating the skin with different microneedle devices after the application of the curcumin formulations caused a tremendous increase in the dermal penetration efficacy of curcumin and showed that not only the size of the curcumin particles in the formulation but also the type of the microneedle device has an impact on the dermal penetration efficacy of curcumin (Figure 6 and Figure 7).

In comparison to the bulk suspension and the skin without microneedle treatment, the amount of penetrated curcumin increased about 2-fold, and the MPD was about 3-fold deeper when the skin was treated with bulk material and the 0.25 mm pen. Treating the bulk-suspension-treated skin with the 0.5 mm device increased the amount of penetrated curcumin about 5-fold, and the MPD was increased almost 6-fold. A further increase in needle length to 1.0 mm could not further increase the dermal penetration efficacy of the curcumin from the bulk suspension.

Treating the skin with the curcumin nanosuspension and with the 0.25 mm microneedle device increased the amount of penetrated curcumin 8-fold and the MPD about 11-fold when compared with the bulk-suspension-treated skin without microneedle treatment. Treatment with the 0.5 mm microneedle device was less efficient than the treatment with the 0.25 mm device, and the treatment with the 1.0 mm device led to a slightly higher amount of penetrated curcumin but a slightly lower MPD when compared with the skin treated with the nanosuspension and the 0.25 mm microneedle device. Hence, the most efficient penetration was achieved when the skin was treated with the nanosuspension and with the 1.0 mm pen. However, the treatment with the 0.25 mm device was almost similarly efficient.

The dermal penetration efficacy of curcumin was similar (*p*-value > 0.5) when the skin was treated with the nanosuspension without any microneedle treatment and with the bulk suspension with subsequent 0.25 mm microneedle pen treatment. Treating the skin with the 0.5 mm device showed only minor differences in the dermal penetration efficacy of curcumin between the nanosuspension and the bulk suspension. The amount of penetrated curcumin was 19% higher than that of the nanosuspension, and the MPD was 11%. When the skin was treated with the 1.0 mm device, the total amount penetrated was about 2-fold, and the MPD was 1.6-fold. The largest differences in penetration efficacy were found for the 0.25 mm device, in which the nanosuspension led to a 4.25-fold higher amount of penetrated curcumin and a 3.4-fold deeper MPD when compared with the bulk suspensions.

Differences between the bulk suspension, the nanosuspensions, and the different microneedle devices were also found for the homogeneity index (Figure 7C). The least homogeneous penetration was achieved for the bulk suspension without microneedle treatment. Treating the skin with the nanosuspensions increased the homogeneity by about 10% and treating with the bulk suspension followed by microneedle treatment with the 0.25 mm device yielded a similar increase in homogeneity. The homogeneity index was close to 90% for the skin treated with the bulk suspensions and the 0.5 mm and 1.0 mm devices and was about 90% for all the nanosuspensions and microneedle devices.

The SCT, as a sensitive measure for skin hydration, was not only affected by the size of the curcumin particles (Figure 4D) but also by the treatment with the different microneedle devices (Figure 7D). An increase in SCT indicates an increase in skin hydration, while a decrease indicates a decrease in skin hydration. In this study, an increase in skin hydration is likely to occur due to an increased penetration of curcumin into the SCT, whereas a decrease can be considered to be caused by increased water evaporation of the skin due to the holes created by treating the skin with the different types of microneedles [30].

No differences in SCT were found for the skin treated with curcumin bulk suspension without microneedle treatment and treated with the 0.25 mm and 1.0 mm devices. The SCT of skin treated with the curcumin nanosuspension and the 0.25 mm device was similar to the SCT of skin treated with the bulk suspension without microneedle treatment but slightly thinner than the skin treated with the bulk suspensions and the 0.25 mm device. The SCT of the skin treated with the nanosuspension and the 1.0 mm device was about 15% thinner than the skin treated with the bulk suspensions without needles and with the 1.0 mm needles but showed no difference from the SCT of skin treated with the nanosuspension and the 0.25 mm device. The SCT increased for skin treated with the 0.5 mm device, regardless of whether the bulk or the nanosuspension was applied to the skin.

Results indicate that the use of microneedles tends to decrease skin hydration. This effect was more pronounced for the skin treated with the nanosuspension. The effect was observed for the skin treated with both the 0.25 mm and the 1.0 mm devices. An opposite effect was observed for the skin treated with the 0.5 mm device.

An increase in microneedle length can be considered to create deeper and broader holes in the skin, which can lead to increased transepidermal water loss, i.e., the water that evaporates over time from the skin. The flux of the evaporating water is from inside to the outside of the skin and is opposite to the flux of penetrating molecules that aim to enter the skin from outside to inside. Broader, deeper, and a higher number of holes in the skin will therefore create a greater volume for particles to enter the skin. However, on the other hand, the increased water flux from the inside, along with increased hole size, depth, and number of holes, counteracts this penetration-enhancing effect and might even overwrite it.

**Figure 8:**
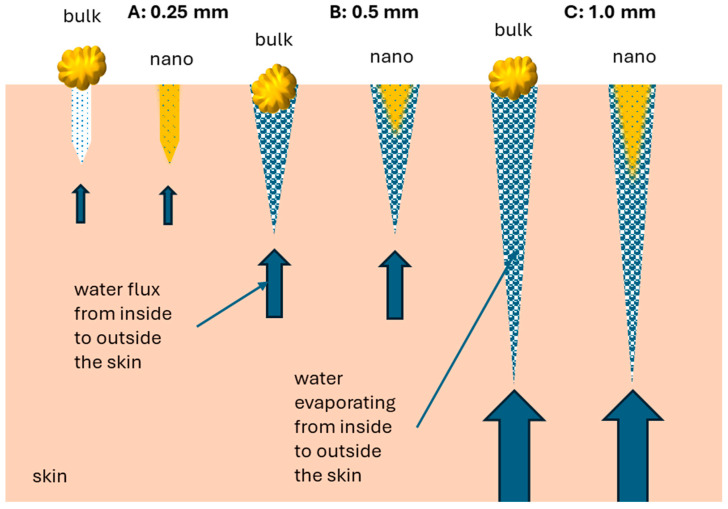
Suggested principle of dermal penetration enhancement of materials from micrometer-sized bulk suspensions and nanosuspensions with different types of microneedles.

The results of this study demonstrate this. Treating the skin with the 0.25 mm device had only a small effect on the dermal penetration efficacy of curcumin from the bulk suspension but allowed for massive penetration of curcumin from the nanosuspension. The small and short needles of the 0.25 mm device created many small holes in the skin (Figure 8A). The holes were too small to allow the large-sized bulk material to penetrate into the skin but were large enough to allow for efficient penetration of the nanocrystals, whose MPD (233 μm) was almost as deep as the needle length (250 μm). The deep penetration of the nanocrystals, which was close to the needle length of the device, indicates that the counteracting water flux from inside the skin was minimal and did not hinder the penetration of the nanoparticles.

Treatment of the skin with the 0.5 mm devices created larger and deeper holes in the skin. The holes were now also large enough for the bulk material. However, the larger size of the holes also increased the water flux and thus hindered the nanocrystals from entering the skin as efficiently as it was possible with the smaller holes from the 0.25 mm device (Figure 8B). A further increase in needle length and number (cf. Figure 1) created slightly more and 2-fold deeper holes and thus increased the water flux from inside the skin. The penetration of the curcumin particles from the bulk material seemed to be hampered by this increased water flux and therefore caused a slightly less efficient penetration when compared with the treatment with the 0.5 mm device (Figure 8C). The nanocrystals, when treated with the 1.0 mm device, showed a higher penetration and deeper penetration when compared with the 0.5 mm device. This result might be a superposition of both effects. The water flux acted against the dermal penetration, but the increased depth of the holes seemed to allow the nanocrystals to penetrate and diffuse more deeply into the skin. As the curcumin itself has a hygroscopic effect, the flux of the water outside the skin might have been decreased by the increasing amount of curcumin inside the skin.

The results confirmed the efficacy of microneedles to improve the dermal penetration efficacy of active compounds from previous works [24,26,27,42,43,44,45,46,47] and showed that the use of microneedles can be very efficient in increasing the dermal penetration of poorly soluble active compounds. The results demonstrate that microneedles allow the particles to enter the skin and show that both the size of the particles and the lengths and types of needles influence the penetration efficacy. Small particles in the submicron range can enter small holes, and the results demonstrated that even very short needles with a length of only 250 μm allowed for an MPD that is larger than the epidermis thickness, which is between 100 and 110 μm [11]. Hence, the submicron particles, in combination with the 0.25 microneedle device, allowed for efficient transdermal penetration, i.e., penetration through the epidermis into the viable dermis. Large bulk particles in the micrometer range, in combination with the 0.25 microneedle device, showed an MPD below 100 μm. Hence, the micrometer particles, in combination with the 0.25 microneedle device, did not allow for efficient transdermal penetration.

**Figure 9:**
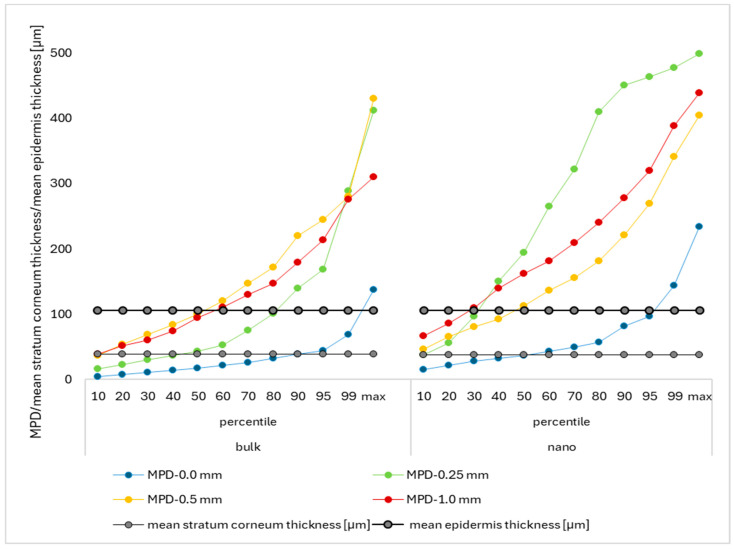
Comparison MPD for skin treated with curcumin bulk material (**left**) and curcumin nanosuspensions (**right**), allowing for an evaluation of the percentage of how much curcumin penetrated through the stratum corneum from the different formulations treated with the different types of microneedles.

However, when comparing the percentiles of the MPD with the mean stratum corneum thickness and the mean epidermis thickness, it becomes obvious that, even if the MPD is below the mean epidermis thickness, some of the particles will enter the viable dermis (Figure 9).

For the bulk material, about 20% of the images showed transepidermal penetration of curcumin when the skin was treated with the 0.25 mm device, and for the 0.5 mm and 1.0 mm devices, even more than 40% of the images showed transepidermal penetration for curcumin. For the nanosuspensions, skin treatment with microneedle devices led to transepidermal penetration of curcumin, i.e., MPD > mean epidermis thickness, in more than 90% of the skin images obtained (Figure 9). This means all microneedle devices used in this study allowed for transepidermal delivery of the active compound into the skin, and most likely, all other components of the formulations were also able to penetrate into the skin and reach the viable dermis. This fact can be considered important and should be taken into account when using microneedles and topical products that might contain non-physiological ingredients (e.g., preservatives, perfumes, or mineral oils) that are not intended to enter the viable dermis. Therefore, when using microneedle devices—to avoid non-physiological ingredients from entering the skin—only formulations that contain skin-friendly and non-toxic ingredients should be used. This could be achieved by using sterile products without preservatives that contain only water and compounds that are intended to be in the skin. The use of classical skin care products that contain preservatives and perfumes in combination with microneedles, however, should be avoided.

## 4. Conclusions

Curcumin bulk suspensions that contained curcumin microparticles of about 20 µm and curcumin nanocrystals with sizes of about 250 nm were produced in this study, and the dermal penetration efficacy of curcumin from the different formulations was studied when applied without and with different types of microneedle devices. The results proved that microneedles increased the dermal penetration efficacy of curcumin efficiently and allowed for transepidermal drug delivery. Both the size of the particles and the lengths of the needles had an impact on the dermal penetration efficacy. Small-sized nanocrystals were most effective, and the use of very short needles with only 250 µm needle lengths allowed for highly efficient transepidermal penetration of curcumin. From the data, it can be concluded that the dermal application of nanocrystals combined with microneedles represents a promising advancement in dermal drug delivery for poorly water-soluble drugs.

## Figures and Tables

**Table 1:** Overview of particle sizes of curcumin suspensions.

Size Parameter	Curcumin Bulk Suspension			Curcumin Nanosuspensions		
z-average [nm]	n.a.	±	n.a.	256	±	13
PDI	n.a.	±	n.a.	0.31	±	0.06
d(v) 0.1 [μm]	4.73	±	0.02	0.02	±	0
d(v) 0.5 [μm]	18.5	±	0.1	0.06	±	0.01
d(v) 0.9 [μm]	47.5	±	0.3	0.51	±	0.1
d(v) 0.95 [μm]	57.9	±	0.4	1.63	±	0.6
d(v) 0.99 [μm]	76.6	±	0.7	3.8	±	0.3

## Data Availability

The authors confirm that the data supporting the findings of this study are available within the article and its Supplementary Materials.

## Supplementary Materials

The following supporting information can be downloaded at: https://www.mdpi.com/article/10.3390/mi16020155/s1, Table S1: macro used for the automated RGB threshold for the digital image analysis.
