# Supplementary material for: Microneedle-Assisted Delivery of Curcumin: Evaluating the Effects of Needle Length and Formulation
Source: Micromachines (Basel). 2025 Jan 29;16(2):155. doi: 10.3390/mi16020155 (PMC11857367; doi:10.3390/mi16020155)
Supplement: Supplementary file 1 [file micromachines-16-00155-s001.zip › micromachines-3443472-supplementary.pdf]

Microneedle-Assisted Delivery of Curcumin: Evaluating the Effects of Needle Length and Formulation

Em-on Chaiprateep [1,2], Soma Sengupta [1] and Cornelia M. Keck [1,*]

[1] Department of Pharmaceutics and Biopharmaceutics, Philipps-Universität Marburg, Robert-Koch-Str. 4, 35037 Marburg, Germany; emon_c@rmutt.ac.th (E.-o.C.); sengupts@pharmazie.uni-marburg.de (S.S.)
[2] Faculty of Integrative Medicine, Rajamangala University of Technology Thanyaburi (RMUTT), Thanyaburi 12130, Thailand
* Correspondence: cornelia.keck@pharmazie.uni-marburg.de; Tel.: +49-(0)-6421-2825881

Table S1: Macro used for the automated threshold to subtract the autoflorescence of the skin from the fluorescence of the curcumin penetrated

```
// Color Thresholder 1.53a
// Autogenerated macro, single images only!
min=newArray(3);
max=newArray(3);
filter=newArray(3);
a=getTitle();
run("RGB Stack");
run("Convert Stack to Images");
selectWindow("Red");
rename("0");
selectWindow("Green");
rename("1");
selectWindow("Blue");
rename("2");
min[0]=0;
max[0]=0;
filter[0]="stop";
min[1]=100;
max[1]=255;
filter[1]="pass";
min[2]=0;
max[2]=0;
filter[2]="stop";
for (i=0;i<3;i++){
  selectWindow(""+i);
  setThreshold(min[i], max[i]);
  run("Convert to Mask");
  if (filter[i]=="stop")  run("Invert");
}
imageCalculator("AND create", "0","1");
imageCalculator("AND create", "Result of 0","2");
for (i=0;i<3;i++){
  selectWindow(""+i);
  close();
}
selectWindow("Result of 0");
close();
selectWindow("Result of Result of 0");
rename(a);
// Colour Thresholding-------------
run("Invert");
```
